# Overview of Health-Monitoring Technology for Long-Distance Transportation Pipeline and Progress in DAS Technology Application

**DOI:** 10.3390/s24020413

**Published:** 2024-01-10

**Authors:** Yuyi Wu, Lei Gao, Jing Chai, Zhi Li, Chenyang Ma, Fengqi Qiu, Qiang Yuan, Dingding Zhang

**Affiliations:** 1 China Coal Energy Research Institute Co., Ltd., Xi’an 710054, China; wuyuyi@chinacoal.com; 2 College of Energy Engineering, Xi’an University of Science and Technology, Xi’an 710054, China; gaolei@stu.xust.edu.cn (L.G.); chaij@xust.edu.cn (J.C.); cheny@stu.xust.edu.cn (C.M.); qfq2021@stu.xust.edu.cn (F.Q.); 3 School of Mines, China University of Mining and Technology, Xuzhou 221116, China

**Keywords:** long-distance transportation pipeline, influence factor, health-monitoring technology, DAS, spatialization

## Abstract

There are various health issues associated with the different stages of long-distance pipeline transportation. These issues pose potential risks to environmental pollution, resource waste, and the safety of human life and property. It is essential to have real-time knowledge of the overall health status of pipelines throughout their entire lifecycle. This article investigates various health-monitoring technologies for long-distance pipelines, providing references for addressing potential safety issues that may arise during long-term transportation. This review summarizes the factors and characteristics that affect pipeline health from the perspective of pipeline structure health. It introduces the principles of major pipeline health-monitoring technologies and their respective advantages and disadvantages. The review also focuses on the application of Distributed Acoustic Sensing (DAS) technology, specifically time and space continuous monitoring technology, in the field of pipeline structure health monitoring. This paper discusses the process of commercialization development of DAS technology, the main research progress in the experimental field, and the open research issues. DAS technology has broad application prospects in the field of long-distance transportation pipeline health monitoring.

## 1. Introduction

Long-distance pipeline transportation is a technology that utilizes pressure to transport gas, liquid, or solid materials to their destination by constructing pipelines over long distances, either on the ground or underground. According to the type of materials being conveyed, the length of the pipeline can range from tens to thousands of kilometers. As vital components of the energy system, pipelines serve as the “blood vessels” that ensure the safe transmission of national energy and contribute to the stable and sustainable development of the economy [1]. Pipeline transportation has several advantages, including large transportation capacity, reduced vulnerability to external environmental factors, long transmission distance, good sustainability, and low cost. As a result, it is extensively utilized in the transportation field [2]. However, the pipeline itself is susceptible to various factors such as damage, aging, cracking, corrosion, transportation medium, and human factors. At the same time, as the pipeline service time continues to increase, certain sections of the pipeline are susceptible to pipe wall thinning and material aging. This can result in the loss of functionality in pipeline transportation, leading to abnormal occurrences such as leakage, blockage, and other conditions [3]. The abnormal transportation of pipelines not only causes environmental pollution and resource waste but also endangers human life and property safety [4]. Therefore, it is necessary to implement advanced technological means to achieve real-time monitoring of abnormal situations in pipeline transportation. This is crucial for ensuring the safety and efficiency of pipeline transportation.

In the early days, manual visual inspection, periscope monitoring, and other methods were often used to monitor the condition of pipelines. The use of simple tools for pipeline monitoring offers the advantages of convenience, speed, and low cost, making them widely used in pipeline inspections. However, monitoring of the pipeline is primarily focused on post-accident or post-problem situations, resulting in a certain degree of lag [5]. In addition, traditional methods of monitoring pipelines heavily rely on the staff’s work experience, and most of the monitoring equipment used is expensive and not easily portable [6]. With the continuous progress of monitoring technology, pipeline monitoring has transitioned from manual to intelligent machines. At present, the main methods for pipeline monitoring at home and abroad include the pipeline closed circuit television method [7], the negative pressure-wave method [8], infrared thermal imaging [9], and some methods that rely on acoustic principles for monitoring. However, these methods have limitations, such as limited measurement distance, high equipment prices, and the possibility of missing inspections. In addition, as the total mileage of the pipeline continues to increase, it becomes particularly important to monitor the overall transportation status of the pipeline [10]. It is challenging to achieve real-time monitoring of long-distance pipeline transportation status using traditional pipeline-monitoring methods. Therefore, there is an urgent need for a long-distance, fully covered, and real-time pipeline monitoring and measurement scheme that can identify and warn of abnormal situations in pipeline transportation in real time, as well as locate abnormal locations.

Distributed Fiber Optic Sensing (DFOS) technology has several advantages, including long-distance sensing, a strong anti-interference ability, good continuity, and strong real-time performance [11]. It can perceive multiple parameters such as deformation, temperature, vibration, etc. It has gradually become widely applied in various fields, including pipeline transportation, roads and bridges, steel structures, and geotechnical and geological engineering. It has also emerged as one of the important technologies in pipeline transportation monitoring [12]. Distributed fiber optic-sensing technology can be divided into two categories based on principles: scattering and interference [13]. The fiber optic-sensing technology, based on the scattering principle, can be divided into three categories according to the different types of backscattered light: Rayleigh scattering, Brillouin scattering, and Raman scattering [14]. In various types of distributed fiber optic-sensing monitoring, fiber optic-distributed acoustic sensing (DAS) technology, which is based on backward Rayleigh scattering, is increasingly being used for pipeline transportation status monitoring [15].

This article first discusses the factors that affect the structural health of pipelines during long-distance transportation. It then analyzes the characteristics of abnormal pipeline health under different influencing factors. Secondly, in response to the aforementioned pipeline health issues, we discussed the main methods currently used for pipeline health monitoring and summarized the advantages and disadvantages of each method. Then, the current research status of fiber optic-sensing technology in pipeline health monitoring was discussed, along with the development status of DAS-monitoring technology and its progress in research on pipeline transportation health monitoring. Finally, the potential applications of DAS-monitoring technology in the field of pipeline health monitoring were proposed.

## 2. Factors and Characteristics Affecting Pipeline Health

Due to the gradual increase in the mileage of long-distance pipelines, the transportation of various objects, significant variations in the external environment, and the increasing service time each year, the types of pipeline accidents have become more diverse. This presents challenges for pipeline health monitoring. Various countries conduct statistical analyses on the causes and types of pipeline accidents. The United States primarily compiles information on pipeline accidents through the Pipeline and Hazardous Materials Safety Administration (PHMSA). The department believes that the common causes of pipeline accidents currently include seven categories: corrosion, excavation damage, mis-operation, material/welding/equipment failure, damage from natural forces, and damage from other external forces, among other reasons. Canada categorizes the causes of pipeline failure into six categories: metal loss, cracking, external effects, materials/manufacturing/construction, and geological disasters, among other causes [16]. This article combines existing classifications and categorizes the factors that impact pipeline health into internal and external factors. It does so from the perspective of selecting appropriate pipeline health-monitoring technologies [17]. Among them, internal factors mainly arise from the impact of the pipeline transportation process on pipeline health. These factors include material defects, pipeline corrosion, and aging damage. External factors primarily arise from the influence of external environmental interference on pipeline health. These factors include human factors (such as third-party human damage, intentional damage, and improper operation during construction and maintenance), as well as natural and geological disaster factors. The statistics of factors affecting the health of long-distance transportation pipelines are shown in Table 1.

The health problems of pipelines caused by internal factors are mostly due to the accumulation of structural damage during long-term transportation. When damage accumulates to a certain extent, it can cause pipeline leakage or blockage. The corresponding abnormal characteristics of pipeline transportation are primarily manifested as concealment, slowness, hysteresis, and randomness. When selecting monitoring methods for pipeline health issues caused by influencing factors, it is important to consider whether the chosen method can provide long-term monitoring of the entire pipeline and accurately identify areas with health abnormalities. The health problems of pipelines caused by external factors are mostly transient damages to the pipeline structure caused by external forces during normal transportation. These damages are characterized by easy identification, instantaneity, large deformation, and predictability. For most human factors, such as improper operation, measures such as enhancing personnel training, improving management of the pipeline’s surrounding area, and establishing warning lines can be implemented to minimize or prevent their impact on pipeline transportation as much as possible. Natural and geological disasters, as well as intentional damage caused by a few human factors, require monitoring and early warning of macroscopic deformation of pipelines, as well as the accurate positioning of accident points through monitoring technology.

## 3. Overview of Pipeline Health-Monitoring Technology

Pipeline-monitoring technology can be divided into five categories based on principles: visual, electromagnetic, acoustic, optical, and chemical-composition monitoring [18]. The corresponding technical refinement and classification of the five types of pipeline status-monitoring methods are shown in Table 2.

### 3.1. Visual-Monitoring Technology

The use of visual-monitoring technology to monitor pipelines, where workers utilize optical images to assess the condition of pipelines for pipeline monitoring, offers the benefits of cost-effectiveness and clear visualization. As a result, it is extensively employed for the internal surface monitoring of pipelines [19].

(1) Closed Circuit Television (CCTV) technology

**Figure 1:**
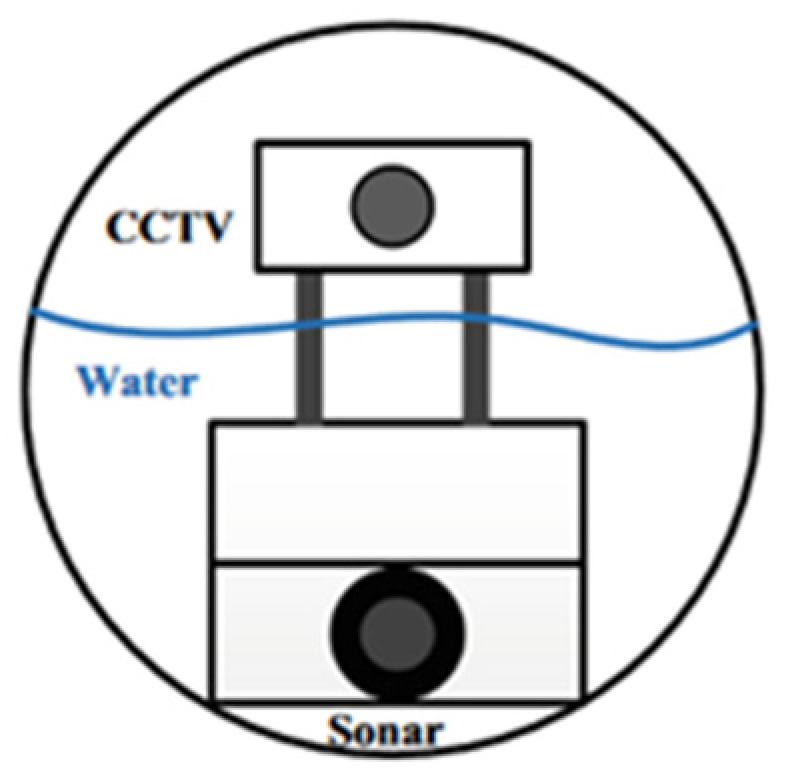
Principle of Combining Closed Circuit Television (CCTV) and Sonar Technology to Monitor Pipelines.

CCTV technology is commonly used for monitoring drainage pipelines. It involves real-time video recording and computer analysis of the pipeline’s condition and transportation status based on the captured images [20]. This type of monitoring technology offers better economy and safety compared to relying solely on manual monitoring of pipelines, but it also has certain limitations. When CCTV monitors pipelines, it can only provide information above the liquid flow line inside the pipeline and cannot achieve quantitative analysis of defects such as pipeline deformation and surface damage [21]. In order to address the limitations of traditional visual-monitoring technology, researchers have turned to computer analysis of pipeline visual-monitoring results to achieve both qualitative and quantitative evaluation of pipeline defects [22]. In addition, the limitations of this technology in pipeline monitoring can be overcome by integrating it with other pipeline-monitoring technologies. For example, if CCTV is used to monitor pipelines with high water levels, which cannot effectively capture underwater conditions, sonar technology can be used as a supplement to scan the pipelines, compensating for the limitations of CCTV pipeline-monitoring technology [23]. The specific combination method is shown in Figure 1.

(2) Side scanner and evaluation technology (SSET) technology

SSET technology, as an alternative to traditional CCTV inspection methods, can accurately display various defects in pipelines by generating detailed digital images through optical scanning and gyroscope technology. With the development of automatic defect-recognition software, the monitoring capabilities of CCTV and SSET technology are becoming increasingly powerful. Based on SEET technology, Chae et al. [24] developed a neural fuzzy algorithm for monitoring and classifying pipeline defects. The digital color image is preprocessed to obtain the corresponding grayscale image, which is then segmented to determine the characteristics of pipeline defects.

(3) 5G drone-inspection technology

**Figure 2:**
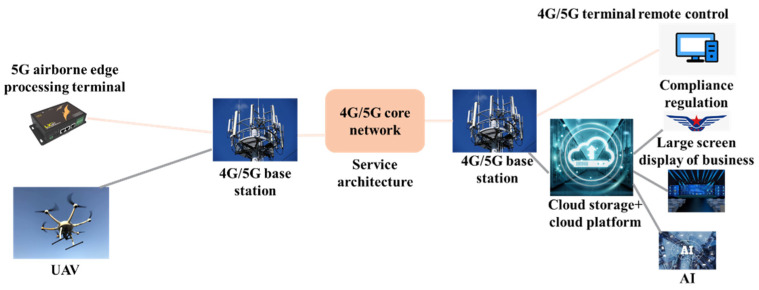
Schematic diagram of 5G unmanned aerial vehicle-inspection architecture design for the Nanjing Zhenjiang section of the Sunan-refined oil pipeline.

5G unmanned aerial vehicle-inspection technology combines mobile internet 5G, unmanned aerial vehicles, and big data analysis technology to achieve unmanned inspection and monitoring of pipeline health. Jiangsu Petroleum Company achieved unmanned inspection and monitoring of an 85 km oil pipeline section by utilizing six 5G base stations and a dedicated 5G low-altitude network built along the pipeline in conjunction with drones. The schematic diagram of its architecture is shown in Figure 2.

### 3.2. Electromagnetic-Monitoring Technology

Electromagnetic-monitoring technology mainly includes various techniques such as magnetic flux leakage, electromagnetic eddy current, broadband electromagnetic, ground-penetrating radar, and pulse eddy current monitoring. The development level of different technologies varies [25]. The following content provides a brief introduction to magnetic flux leakage, electromagnetic eddy current, and ground-penetrating radar-monitoring technology.

1. Magnetic flux leakage technology

**Figure 3:**
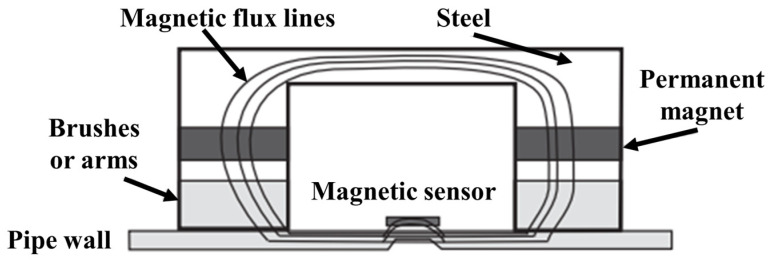
Principle of Magnetic-Leakage Technology for Monitoring Pipeline Leakage.

Magnetic flux leakage technology mainly uses permanent magnets to magnetize the pipe wall. When there are defects in the pipeline, the magnetic field lines will leak onto the surface of the pipeline, resulting in magnetic flux leakage. Subsequently, the magnetic flux-leakage signal is extracted by magnetic-sensing elements in order to detect pipeline defects. The schematic diagram is shown in Figure 3.

2. Electromagnetic Eddy Current Technology

Electromagnetic eddy current technology has several advantages, including strong anti-interference ability, non-contact monitoring, and high sensitivity. It is widely utilized in the monitoring and evaluation of ferromagnetic pipelines [24]. At present, there is a large number of research achievements in the evaluation of pipeline status using far-field eddy current signals generated by electromagnetic eddy current technology. Antipov et al. [26] used finite element analysis to enhance the visualization of monitoring results generated by this method, significantly improving research efficiency. Grimberg et al. found that the monitoring of circumferential and axial defects in pressure pipelines can be effectively achieved using three-phase rotating far-field eddy currents. Yang et al. improved the sensitivity of the sensor for pipeline monitoring by adjusting the structure of the far-field pulse eddy current to create a multi-layer magnetic field, thereby further concentrating the magnetic field energy.

3. Ground-penetrating radar technology

The principle of ground-penetrating radar technology is that the radar emits electromagnetic waves, which will reflect when encountering interfaces or objects during the propagation process. The integrity and status of pipelines are determined by analyzing the waveform, amplitude, and other characteristics of the received electromagnetic waves. This technology has several advantages, including high efficiency, low cost, and non-contact monitoring. It is considered one of the important practical technologies in pipeline non-destructive monitoring. Stampolodis et al. [27] applied ground-penetrating radar technology to monitor urban underground water pipelines. They successfully distinguished different types of pipelines and established a certain understanding of reflection patterns related to pipeline leakage. David et al. focused their attention on feature analysis of ground-penetrating radar images. They extracted features from the corresponding image results in order to achieve the self-localization of plastic material pipelines. This study not only effectively assists in the preprocessing of radar-detection raw data but also proposes the classification of different types of leakage features of plastic pipelines. It has played a significant role in driving the application of ground-penetrating radar technology in pipeline detection. Lai et al. conducted indoor experiments to simulate the leakage of PVC pipes. They detected the leakage using ground-penetrating radar technology and studied the potential interference that leakage signals may have on the detection technology. Their research successfully achieved the monitoring and identification of PVC pipe leakage.

### 3.3. Acoustic-Monitoring Technology

Acoustic-monitoring technology primarily monitors and identifies sound or vibration signals resulting from abnormal conditions in pipelines. When an abnormal situation occurs in the pipeline during transportation, a pressure difference is formed between the inside and outside of the pipeline. This pressure difference further generates turbulence, leading to vibrations near the leakage point and promoting the generation of acoustic signals [28]. There are various types of acoustic-based pipeline-monitoring technologies, and they can be classified into two categories: active monitoring and passive monitoring.

1. Acoustic-emission technology

**Figure 4:**
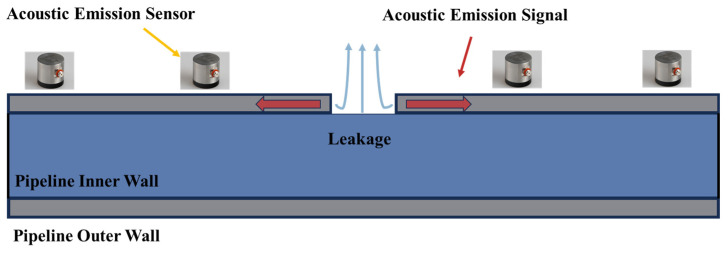
Principle of Pipeline Leakage Monitoring and Location Based on Acoustic-Emission Technology.

Acoustic-emission technology is achieved by monitoring the propagation of elastic waves generated by active cracks, leaks, or impacts. The schematic diagram is shown in Figure 4. This technology is a widely used passive non-destructive evaluation-monitoring technique that enables global and real-time monitoring of pipelines. In addition, it can also achieve defect location positioning. This technology is primarily used for identifying pipeline leakage and damage, with relatively little research focused on pipeline blockage status. Juliano et al. [29] successfully applied acoustic-emission technology in practical pipelines to identify and locate locations of pipeline leakage. Mostafapour et al. utilized acoustic-emission technology to analyze and locate the leakage location of high-pressure pipelines in indoor experiments. They validated this conclusion through mathematical modeling. Strei et al. [30] achieved the accurate positioning of leakage locations in liquid-filled pipelines by increasing the number of sensors and employing triangulation methods. Currently, the primary limitation of acoustic-emission technology is the need for a significant number of sensors to cover the entire length of long-distance transportation pipelines. This requirement increases the cost of pipeline monitoring and makes it impractical for practical engineering purposes.

2. Ultrasonic-monitoring technology

Ultrasonic-monitoring technology is considered one of the important technologies for the non-destructive monitoring of pipelines. It mainly achieves the identification and positioning of discontinuous situations in pipelines through the use of ultra-high frequency acoustic energy [31]. As one of the traditional pipeline-monitoring technologies, ultrasonic monitoring is commonly used to estimate the remaining wall thickness of pipelines offline. However, such measurements are typically conducted during routine shutdowns and maintenance of pipelines, resulting in a slow evaluation speed of pipeline-monitoring results. In addition, the current use of traditional ultrasonic technology for pipeline monitoring mainly involves point-by-point monitoring. In contrast, ultrasonic-guided wave non-destructive monitoring mainly involves monitoring low-frequency torsional or longitudinal waves generated by excitation. These waves can propagate over long distances along the waveguide with minimal signal attenuation. Therefore, compared to traditional ultrasonic-monitoring techniques, ultrasonic-guided wave non-destructive testing technology offers higher sensitivity and is more concise and reliable in monitoring damage and cracks caused by corrosion in pipelines. This technology enables the long-distance monitoring of pipelines [32]. Mao et al. [31] utilized ultrasonic-monitoring technology to emit ultrasonic waves for oil pipelines. They compared and analyzed the ultrasonic signals reflected by damaged and undamaged pipelines and processed the reflected wave signals using HHT (Hilbert–Huang Transform) technology. They successfully achieved the monitoring and identification of discontinuous positions in oil pipelines, demonstrating the feasibility and accuracy of ultrasonic-monitoring technology in pipeline surveillance. Wissam et al. successfully detected and characterized defects at the welding position of pipeline interfaces, validating the use of ultrasonic-monitoring technology for detecting micro damage in pipelines.

3. Impact echo method

The impact echo method has several advantages, such as strong anti-interference, deep penetration, and high resolution. It is a promising technology for monitoring pipeline defects. Usually, it is considered a technology that utilizes the propagation characteristics of stress waves in the medium to monitor internal defects in pipelines. Shi et al. [33] compared and analyzed the current detection methods and practical applications of concrete grouting pipelines. They found that impact echo-monitoring technology has advantages in detecting defects in grouting pipelines. Yang et al. conducted an analysis and study on the factors that influence the accuracy of the impact echo-detection method. They concluded that the material of the pipeline, the severity of pipeline defects, and the location of pipeline defects all have an impact on the detection results of the impact-echo method. Tan et al. [27] used the impact-echo method to detect concrete grouting pipelines and found that this method performs well in identifying internal defects in pipelines.

### 3.4. Optical-Monitoring Technology

1. LiDAR technology

LiDAR (Light Detection and Ranging) is also known as optical radar technology. The working principle of this device is to emit an infrared beam outward and analyze the detected object by receiving signals reflected by it. In terms of pipeline monitoring, traditional manual mapping methods cannot achieve high-precision mapping of complex terrain areas and cannot efficiently obtain accurate information about the pipeline’s condition. In comparison, the long-distance pipeline detection scheme, launched by the Feiyan Remote-Sensing Company, combines aerial photography and LiDAR technology to achieve digital modeling of the surrounding environment and terrain of the pipeline. This approach is beneficial for the subsequent operation, management, and maintenance of the pipeline. Chae et al. [34] believe that when using LiDAR technology to monitor the interior of pipelines, the data-collection method of this inspection technique is relatively limited, and as a result, it is often unable to provide a comprehensive description of the overall condition of the pipeline’s interior.

2. Thermal radiation-imaging technology

Thermal radiation-imaging technology is used to detect the thermal radiation emitted by an object itself by collecting the corresponding light in the thermal infrared band. The collected thermal radiation results are then converted into gray values, allowing for the recognition of the object’s status through gray difference imaging [35].

3. Optical fiber-sensing monitoring technology

Optical fiber-sensing monitoring technology involves transforming traditional optical fiber into a virtual array of sensors, several kilometers in length, through a dynamic measurement system [36]. This is achieved with the help of a corresponding demodulation and analysis scheme, enabling the monitoring and positioning of the full-length acoustic and vibration signals of the optical fiber. Distributed optical fiber-sensing technology has the advantages of long-distance and large-scale monitoring. The widely used technologies can be further divided into three categories: optical time domain reflectance (OTDR) technology, interferometric fiber-sensing technology, and fiber Bragg-grating (FBG) technology. The basic principle of OTDR technology is that when the light emitted by the light source propagates forward along the optical fiber, it will produce backscattering [37]. The intensity of the backscattered light will gradually decay with the increase in distance. When the speed of light is a certain value, the distance is proportional to time. Therefore, by analyzing the intensity of backscattered light detected by the detector and the corresponding time it takes to reach the detector, it is possible to determine the initial backscattered light intensity at any point along the fiber path. This enables the effective monitoring of pipeline transportation.

**Figure 5:**
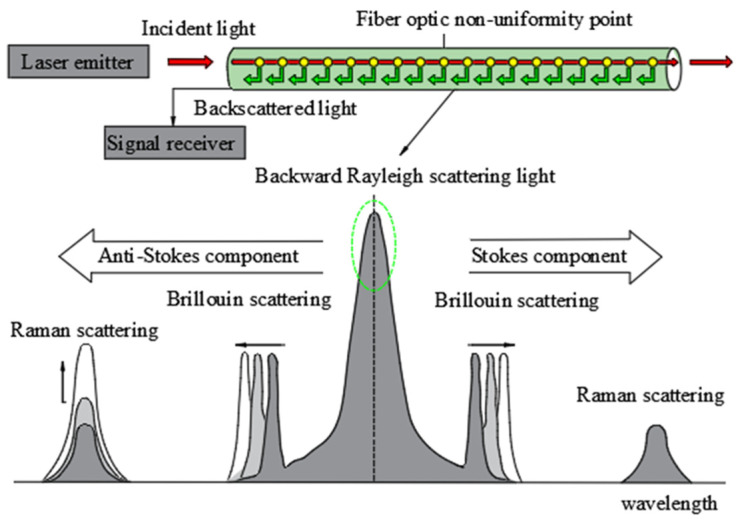
Principle of distributed optical fiber-sensing monitoring.

According to the working principle of OTDR technology, it is based on backscattering. Backscattering can be further divided into Rayleigh scattering, Brillouin scattering, and Raman scattering [38]. Among them, Rayleigh scattering is a phenomenon caused by the inhomogeneity of the refractive index of the optical fiber resulting from the inhomogeneity of its own material. Raman scattering, on the other hand, is caused by the interaction between light and matter molecules, resulting in the production of optical phonons. Brillouin scattering, on the other hand, is caused by the periodic change in the density of the optical fiber material, resulting in the production of acoustic phonons. The three scattering modes in distributed optical fiber-sensing monitoring are shown in Figure 5. It can be seen from the figure that in the backscattering of light, the intensity of Rayleigh scattering is high. When the optical fiber is disturbed by an external physical field, the intensity of backscattered light will decrease significantly. Therefore, it is possible to monitor the disturbance of the optical fiber by measuring the change in backscattered light intensity. Relatively speaking, Brillouin scattering and Raman scattering have low light intensity, which makes them unsuitable for measuring changes in light intensity. However, when considering the relationship between the frequency shift and the changes in temperature and strain, it is common practice to monitor the variations in external temperature, strain, and other factors by measuring the frequency shift in these two types of scattering methods. Gao et al. [39] used an optical time domain reflectometer (OTDR) to monitor the safety of buried oil and gas pipelines, reducing the range for locating damaged points to 22.4 m. Qu Zhigang and his team developed an early warning system for detecting oil and gas pipeline leakage using a Mach Zehnder optical fiber interferometer. They also established a corresponding monitoring system that can identify pipeline leakage and other abnormal conditions. Stajanca and others applied the distributed acoustic-sensor monitoring (DAS) system to monitor the gas leakage in the pipeline.

### 3.5. Discussion on Characteristics of Different Pipeline-Monitoring Technologies

Visual-monitoring technology for pipelines is continuously advancing towards greater accuracy, faster response times, and improved cost-effectiveness. Among them, the primary development trend is to achieve an automatic identification and judgment of data in order to enhance the effectiveness and monitoring efficiency of pipeline-monitoring results. However, this method relies on manual labor, meaning that staff members need to analyze it in order to obtain relevant judgments about the condition of the pipeline. Additionally, it can also cause damage to the structure of the pipeline. Therefore, the accuracy and efficiency of the monitoring technology in the automatic identification of defects still need to be further improved [6]. Electromagnetic monitoring is a non-destructive technology used for monitoring. It offers several advantages, including a simple structure, high accuracy, non-contact monitoring, minimal environmental impact, real-time signal feedback, and timely data acquisition. As a result, it is widely employed for online monitoring of pipelines. However, so far, the application of electromagnetic monitoring in pipelines has mainly focused on monitoring damage, cracks, and other related aspects. Yet further research and development are needed to explore its application in other areas [39]. Acoustic-monitoring technology, which involves monitoring the acoustic signals caused by pipeline abnormalities, includes both passive and active types. This method can achieve the non-destructive detection of the pipeline. However, since it is a point sensor, it requires a large number of equipment to detect the entire length of the pipeline when applied to long-distance pipelines. This method lacks economy and practicality. Optical-monitoring technology enables monitoring by analyzing the reflection or scattering results of different types of light through the measured pipeline. Among the various optical-monitoring technologies, different methods exhibit varying performances in pipeline monitoring. If non-contact optical technologies, such as laser radar and thermal radiation imaging, are selected, they can only assess the condition of the pipeline in a specific area around the monitoring equipment. Additionally, these technologies are susceptible to interference from the external environment. The optical fiber-monitoring technology can effectively solve the problem of detecting the entire length of a long-distance transmission pipeline. However, it is necessary to ensure close contact between the technology and the pipeline being measured.

Pipeline health monitoring requires continuous, real-time monitoring over an extended period to understand its evolving patterns. In addition, monitoring information is time sensitive, and it is usually necessary to rely on the long-term installation of sensing equipment and real-time data collection on the pipeline to identify the transportation status of the pipeline. Based on their ability to achieve continuous monitoring in both time and space, pipeline monitoring methods can be divided into two categories: discontinuous monitoring and continuous monitoring. The discontinuous-monitoring method can only provide feedback on the health status of specific sections of the pipeline or monitoring points in terms of space. It cannot achieve continuous monitoring over time. The continuous-monitoring method enables fully distributed and real-time feedback on the health status of pipelines in both time and space. From the perspective of monitoring data types and processing, this article provides an overview of the data-processing methods used in various monitoring categories. For discontinuous-monitoring methods, the main techniques used include the volume/mass-balance method, the negative pressure-wave method, the GPS time-marking method, and the fiber Bragg-grating wavelength division multiplexing method. For continuous monitoring, optical time domain reflectometry (OTDR) and interferometric optical fiber acoustic-analysis methods are used. Cross-correlation analysis and transient time-frequency analysis methods are applicable to both types of monitoring methods. The classification of pipeline-monitoring methods and data-processing methods is shown in Table 3.

## 4. Research Progress of DAS Technology

**Figure 6:**
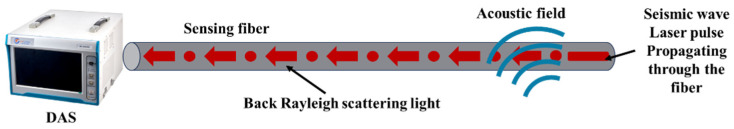
DAS system working process.

DAS (Distributed Acoustic Sensing), or distributed acoustic sensing, is a new technology that enables the continuous and distributed detection of vibrations and sound fields. It utilizes the properties of highly sensitive coherent Rayleigh scattering, which is excited by a narrow linewidth single frequency laser in an optical fiber. This technique combines with the principles of an optical reflectometer to detect environmental vibrations and sound field information that interact with the optical fiber over long distances, with high temporal and spatial accuracy. In general, it is a new technology that obtains acoustic vibrations through an optical fiber sensor. The working process of DAS technology can be simply described as follows: the laser injects pulsed light into the fiber, where a portion of the pulsed light interferes with the incident light inside the fiber. The backscattered interference light is then brought back to the signal processor. At the same time, the vibration information along the fiber will be transmitted back to the signal processor. Based on the relationship between the speed of light and the optical path, the measurement results of fiber vibration at a specific distance can be obtained through calculations. The following Figure 6 shows the working process of the DAS system.

### 4.1. DAS Measurement Principle

**Figure 7:**
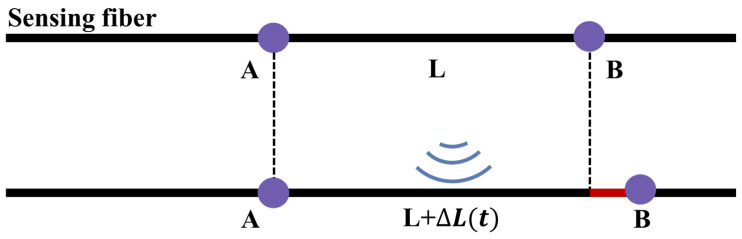
DAS measurement principle.

In DAS, the optical fiber can sense sound waves by measuring the optical phase change caused by the axial strain of the fiber [36]. When the sound wave acts on the fiber, it causes the fiber to strain in the axial direction, thereby altering the phase of the Rayleigh scattering signal within the fiber [40]. The schematic diagram of the measurement is shown in Figure 7 below.

Referring to the DAS measurement schematic diagram shown in Figure 7, it is assumed that there are two points on the optical fiber: Points A and B. The distance between them is L. Suppose that the backscattered signals corresponding to Points A and B have Amplitudes $E_{1}$ and $E_{2}$ respectively, and Phases $\Phi_{1}$, $\Phi_{2}$. However, if the fiber is interfered with between two points, the Phase Difference ΔΦ of the backscattered signal between the two points is directly proportional to the elongation δL of the optical fiber between the two points [41]. The corresponding mathematical relationship is as follows:

(1) $$\Delta \Phi =n_{c}\frac{2\pi }{\lambda_{l}}\delta L=n_{c}\frac{2\pi }{\lambda_{l}}L\epsilon_{zz}$$

where $n_{c}$ represents the refractive index of the fiber core, $\lambda_{l}$ denotes the wavelength of the laser, δL signifies the elongation of the optical fiber, and $\epsilon_{zz}$ represents the average axial strain of the optical fiber over a measured length. According to the provided formula, the strain variation of this section of optical fiber can be estimated by measuring the phase difference of the backscattered signals at Points A and B.

### 4.2. Development of DAS Technology

**Figure 8:**
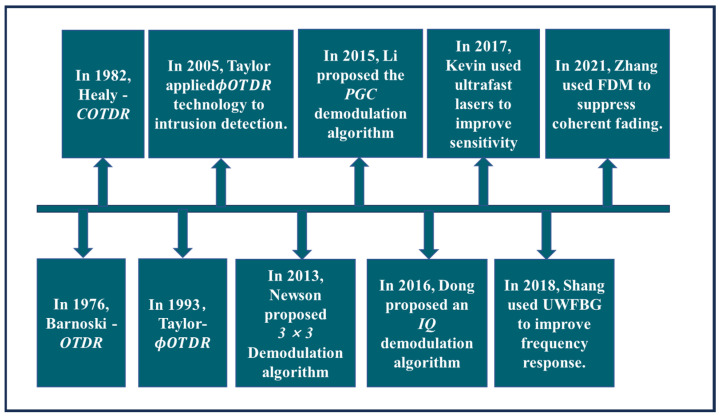
Development history of DAS Technology.

In 1976, Barnoski et al. [42] first proposed optical time domain reflectometry (OTDR) technology based on the design concept of lidar and applied it to monitor optical path loss in optical fiber transmission. Since the OTDR technology cannot respond to the phase-modulation information caused by interference events, i.e., the sensitivity of the signal is not high, Healy et al. [43] proposed the concept of coherent OTDR (C–OTDR) technology in 1982 to further improve the system performance. In 1993, Taylor et al. [44] proposed a highly sensitive phase-sensitive OTDR (Φ−OTDR), DAS began to enter the qualitative-monitoring stage. In order to extract the relevant information from external physical quantities, coherent demodulation technology is proposed to demodulate the internal interference signal. In 2013, Newson et al. [45] proposed a method that utilizes 3 × 3 phase demodulation of a coupler. In 2015, Li et al. [46] proposed a demodulation method for the phase-generated carrier (PGC). In 2016, Rao et al. [47] used the IQ demodulation method to process the optical fiber tensile-deformation signal, which marked the entry of DAS into the field of quantitative monitoring. At present, DAS technology has achieved significant development due to improvements in polarization fading, coherent fading, spatial resolution, frequency response, signal-to-noise ratio, detection range, and other performance indicators [48]. The development history of DAS is shown in Figure 8 below.

### 4.3. Development Status of DAS Technology at Home and Abroad

DAS technology has obvious advantages [49], which can be summarized as the following four points:

1. High-acquisition density and large transmission capacity;
2. Long detection distance and low overall cost;
3. High sensitivity;
4. High-accuracy positioning.

Compared with other distributed-sensing technologies, the advantages of DAS are mainly reflected in two aspects.

1. Efficient real-time monitoring: all detection points in DAS are on the optical fiber, so there is no need to consider the layout and recovery of testing equipment, which can realize the complete length of a single detection, which greatly improves the efficiency and will not affect the normal project when testing data;
2. Good adaptability to the environment: the strong adaptability of the optical fiber to harsh environments makes its maintenance cost low [50]. In addition, optical fiber does not need a separate power supply to achieve multiple repeated monitoring;

Optasense and Silixa in the UK are the first companies to conduct DAS research internationally [51].

At the same time, domestic researchers have also developed some highly sensitive DAS-monitoring systems. In particular, the optical fiber optics research center of Xi’an University of Electronic Science and technology has broken the international record for many times in the monitoring distance and other indicators of DVS/DAS hardware technology based on Φ−OTDR. The parameter comparison of corresponding equipment is shown in Table 4 below.

## 5. Research Progress of DAS-Monitoring Pipeline-Transportation Anomalies

**Figure 9:**
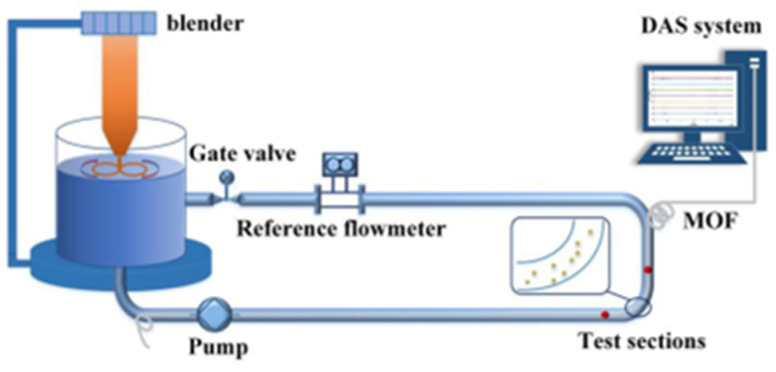
Experimental device for sediment content based on DAS system.

**Figure 10:**
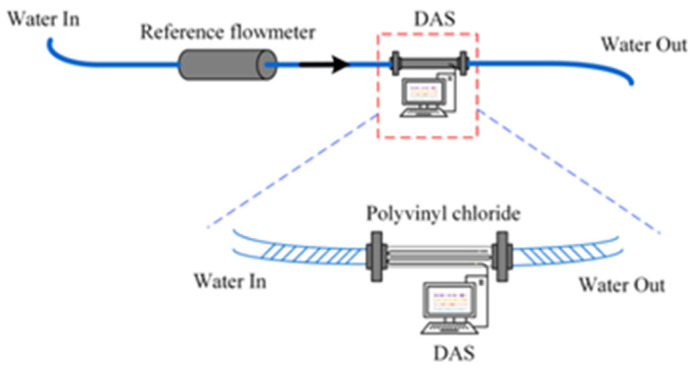
Experimental device of distributed acoustic sensor based on flow-induced vibration.

At present, the current research on pipeline-health monitoring based on Distributed Acoustic Sensing (DAS) primarily focuses on indoor experiments and engineering trials. Li et al. [52] designed an experimental device for measuring sediment concentration. The device includes a solid–liquid two-phase flow-circulation system and a sensing system, which enables real-time monitoring of the sediment concentration in the flow using DAS technology. The experimental device is shown in Figure 9 below. The team improved the strength of the backscattered signal by installing continuous micro-optical fiber structures along the axial direction of the optical fiber. They also amplified the signal of sand collision by utilizing the elbow structure. The experimental results show that this method has the potential for application in real-time monitoring of sediment concentration in long-distance, non-invasive pipelines. The team also designed a distributed acoustic sensor based on flow-induced vibration to monitor pipeline flow. The system diagram is shown in Figure 10 below.

**Figure 11:**
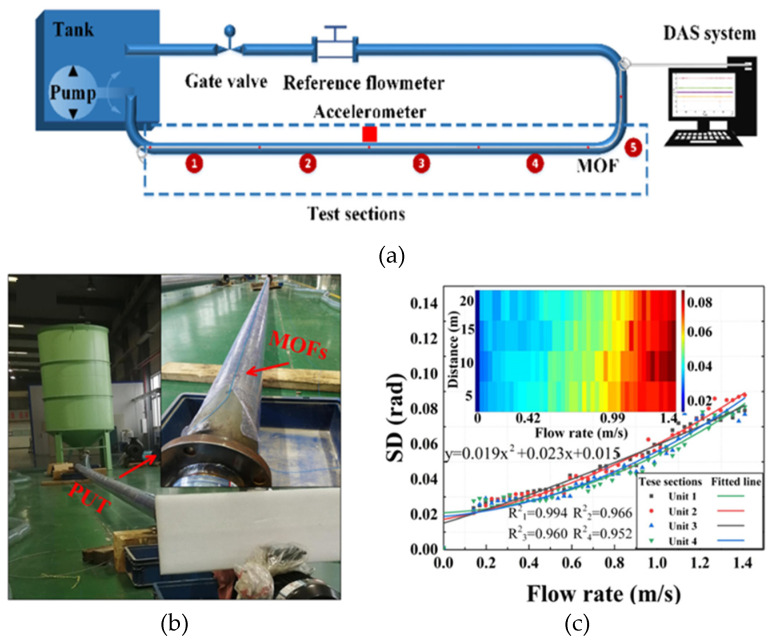
Pipeline flow-monitoring system based on DAS and flow-induced vibration principle. (**a**) The Non-invasive online flow-monitoring experimental device based on DAS system and FIV. (**b**) Field trials. (**c**) Distributed flow-monitoring results of straight pipe sections.

The final results show that there is a quadratic relationship between the flow velocity and the standard deviation of the noise signal caused by flow-induced vibration [53]. In addition, the team utilized a novel distributed microstructure optical fiber acoustic sensor (MOF–DAS) to achieve the monitoring of pipeline flow through the Flow-induced Vibration (FIV) principle. This research is considered to be the first time that Distributed Acoustic Sensing (DAS) has been applied to non-invasive pipeline-flow monitoring. The system diagram is shown in Figure 11 below.

**Figure 12:**
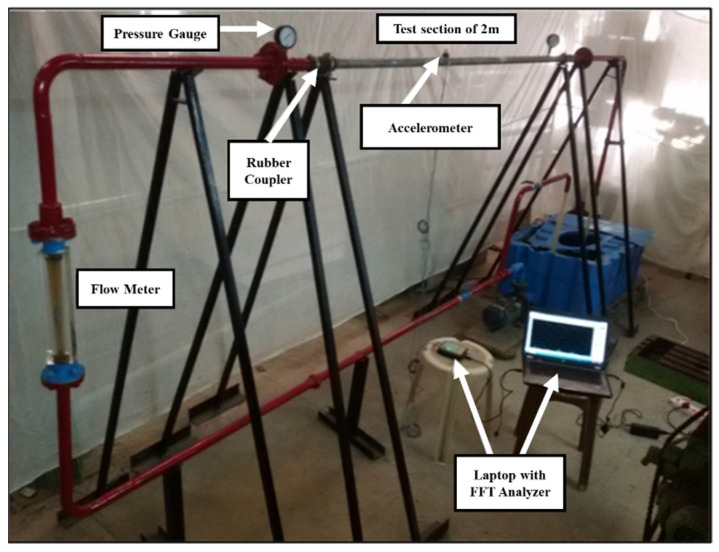
Experimental device for flow-induced vibration of straight pipe.

Wu et al. [54] conducted a study on the hydrostatic leak test of water-transmission pipelines using Technology A. The sensing optical fiber is installed along the inner wall of the pipeline. It collects and analyzes the vibration signal caused by leakage at one end of the fiber to monitor and locate the position of the leak. The test results show that the distributed acoustic sensor has a good response for B steel pipe when the aperture is C and the internal pressure reaches D. Khot et al. [55] conducted an experiment on straight pipe flow-induced vibration using DOE technology. The aim was to investigate the impact of different parameters, such as pipe diameter, pipe-wall thickness, and volume flow, on the vibration amplitude of turbulent straight pipes. Finally, it is concluded that the vibration amplitude of the pipeline increases with the increase in volume flow and slightly increases with the increase in pipeline diameter. The experimental device is shown in Figure 12 below.

**Figure 13:**
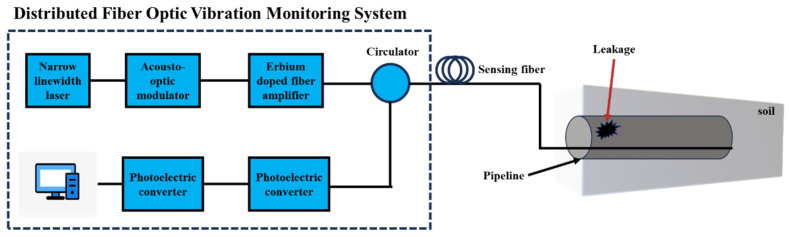
Distributed optical fiber vibration-sensing experimental system for pipeline leakage monitoring.

Liu Zhiwei [56] built a pipeline leakage-monitoring system based on a distributed optical fiber vibration sensor relying on the outdoor experimental platform of Shandong Laser Research Institute and improved the accuracy of the monitoring system in pipeline leakage monitoring to a certain extent with the help of a multidimensional spatial data fusion algorithm. The system diagram is shown in Figure 13 below.

## 6. Summary and Outlook

This review provides readers with a reference to select suitable methods for monitoring pipeline health in various application environments. This paper summarizes the current issues related to pipeline health and discusses various methods for monitoring pipeline health. The influencing factors of pipeline health are divided into two categories: internal factors and external factors. The pipeline-health-monitoring methods are divided into two categories: the discontinuous-monitoring method, which uses local pipelines as the monitoring object, or the health monitoring time is discontinuous; and the continuous-monitoring method, which can realize the full distributed and real-time health monitoring of the whole pipeline. In this section, we will discuss the research progress of DAS technology, which enables continuous pipeline monitoring. We will also highlight the significant application potential of DAS technology in pipeline-health monitoring. The pipeline-health monitoring, based on DAS technology, can ensure the safe and stable operation of pipelines and has promising application prospects. The follow-up application of DAS technology in pipeline-health monitoring should pay attention to the following aspects:

At present, there are limited reference cases for DAS technology in feature-signal recognition and positioning. Therefore, it is still necessary to conduct indoor experimental research. The time-frequency domain representation of the characteristic signal of DAS is influenced by various factors, including the pipeline-installation mode, transmission object, transmission pressure, flow rate, and more. This significantly increases the workload of establishing the characterization test for the characteristic signal of DAS. At the same time, the data characteristics of DAS-monitoring technology are dynamic and involve a large amount of data. This requires the assistance of machine learning or deep-learning methods to effectively denoise the DAS monitoring data and automatically capture characteristic signals. The intelligent processing method of DAS data is also a focal point for future research work.

1. Continuous research on the pipeline layout process of optical fiber sensors is necessary to achieve accurate perception of DAS technology for pipeline abnormal data. When Distributed Acoustic Sensing (DAS) technology is used to monitor the health status of pipeline transportation, the layout of optical fiber directly affects the signal-to-noise ratio of the fiber. This ratio plays a key role in the accuracy and stability of the monitoring results;
2. It is important to ensure that the optical fiber remains undamaged during the layout process, and that the laid optical fiber can effectively couple with any changes in the pipeline. In order to improve the signal-to-noise ratio of the optical fiber, the layout of the optical fiber sensor should be improved in the following aspects: (a) In terms of the layout direction of optical fiber sensor, the distributed optical fiber belongs to the axial strain sensor, which has unidirectionality. When laying optical fibers, they should be laid according to the extension direction of the pipeline structure to ensure that the vibration of optical fibers and pipelines can be coordinated to the greatest extent. (b) Select the appropriate adhesive or fixing method to ensure good deformation coupling between the pipeline and the optical fiber sensor so as to accurately sense the deformation or vibration state of the pipeline. The optical fiber-monitoring system includes three parts: the contact section between the optical fiber sensor and the pipeline, the free section of the optical fiber sensor, and the connection section between the optical fiber sensor and the monitoring equipment. Bending loss shall be avoided during the integration of the optical fiber sensor into the monitoring equipment terminal, and the overall connectivity of the optical path in the optical fiber shall be verified in advance. At the same time, we should pay attention to the deployment technology of the staff, strengthen the late protection measures of the optical fiber sensor, and ensure the smooth implementation of the subsequent experiments;
3. The identification of pipeline-health problems and the spatial positioning of problem areas require Das to have comprehensive diagnostic abilities for pipeline anomalies. In general, the combined use of multiple monitoring methods can ensure the detection of various pipeline-health issues, including pipeline leakage, blockage, and man-made damage. However, this approach inevitably leads to increased monitoring costs and workload while reducing monitoring efficiency. At the same time, the availability of multi-source monitoring data has posed greater challenges to the accuracy of result analysis. Therefore, it is necessary to conduct more laboratory tests on the DAS characterization of pipeline health characteristics, establish a DAS characterization data-model library for different pipeline-health problems so as to realize the identification of different kinds of pipeline-health problems, and finally realize the real-time evaluation of pipeline health with the further research.

## Figures and Tables

**Table 1:** Factors and characteristics affecting the health of long-distance transportation pipelines.

Classification	Influence Factor	Characteristic
Internal factors	Material defects: pipe defects, weld defects, et al.	Concealment Slowness Lag Randomness
	Pipeline corrosion: internal corrosion, external corrosion, hydrogen-induced cracking, et al.	
	Aging damage: natural aging, fatigue damage aging, et al.	
Externalities	Third-party man-made damage: construction, construction, arable land, et al.	Easy to identify Transient Large deformations Predictability
	Intentional destruction: drilling holes to steal oil/gas, illegal occupation and pressure, terrorist activities, et al.	
	Natural and geological disasters: landslides, earthquakes, floods, et al.	
	Improper operation: equipment/control system malfunction, construction damage, improper installation, incorrect operation, improper maintenance, et al.	

**Table 2:** Classification of Common Techniques for Pipeline Monitoring.

Classification	Monitoring Technology
Visual-monitoring technology	Closed circuit television (CCTV) technology, side scanner and evaluation technology (SSET) technology, PANORAMO^®^ 3D technology, drone-inspection technology, et al.
Electromagnetic-monitoring technology	Magnetic flux leakage (MFL) technology, electromagnetic eddy current technology, broadband electromagnetic technology, ground penetrating radar technology, et al.
Acoustic-monitoring technology	Acoustic emission technology, ultrasonic technology, ultrasonic guided-wave technology, impact echo technology, smart ball technology, et al.
Optical-monitoring technology	Lidar technology, diode laser absorption technology, thermal radiation-imaging technology, spectral-imaging technology, fiber optic-sensing technology, et al.
Chemical composition-monitoring technology	Sniffing method, steam-sampling method, et al.

**Table 3:** Classification of pipeline-monitoring and data-processing methods.

Data-Processing Methods	Pipeline-Monitoring Methods
Volume/mass-balance method Negative pressure-wave method GPS time stamping Pressure-point analysis State-estimation method FBG WDM usage	Discontinuous-monitoring methods
Optical time-domain reflectance analysis	Continuous-monitoring methods
Interferometric fiber optic acoustic analysis	
Cross-correlation analysis	Both apply
Transient time-frequency domain analysis	

**Table 4:** Comparison of DAS Equipment Parameters at Home and Abroad.

Company	Products	Detection Range/km	Minimum Measurable Strain	Frequency Response Range/Hz	Characteristics
Silixa	iDAS	40	30 $p\varepsilon /\sqrt{Hz}$@10 Hz	0.01–50,000.00	High low-frequency response
	Carina	25	3 $p\varepsilon /\sqrt{Hz}$@10 Hz	--	Scattering-enhanced fiber optics have high signal quality
Optasense	ODH4	10	--	5.00–200,000.00	Seismic detection
	ODH–F	--	--	5.00–50,000.00	Pipeline-fluid monitoring
	ODH–M	10	--	5.00–100,000.00	Marine-monitoring applications
Fotech	Helios	50	--	5.00–20,000.00	Pipeline applications
Optics Valley Interconnection	Finder	100	--	--	Optical cable routing is accurately positioned, portable and dexterous, and ultra-cost-effective
	Scounter	50	3.4 $p\varepsilon /\sqrt{Hz}$@ ≥ 10 Hz	0.01–50,000.00	Sonic high-fidelity restoration, 0.01 Hz ultra-low frequency response, high sensitivity
	Thinker	50	3.4 $p\varepsilon /\sqrt{Hz}$@ ≥ 10 Hz	0.01–50,000.00	Accurate event identification
CNPC Aobo	uDAS	40	18 $p\varepsilon /\sqrt{Hz}$@15 Hz	1.00–10,000.00	Oil and gas development has a high degree of utilization
Puniu Technology	Hifi–DAS	100	1 $n\varepsilon /\sqrt{Hz}$@5 Hz	5.00–20,000.00	Based on TGD–OFDR, high fidelity
Huawei	--	100	--	--	High-accuracy event recognition

## Data Availability

Data are contained within the article.

## References

[1] Xing W., He X., Yuan L. (2022). Evaluation on the importance of global pipeline natural gas trade node. J. Ind. Technol. Econ..

[2] Qian J., Niu C., Du W. (2021). Development trend and Prospect of Pipeline Intelligent Management. Oil Gas Storage Transp..

[3] Adegboye M.A., Fung W.K., Karnik A. (2019). Recent advances in pipeline monitoring and oil leakage detection technologies: Principles and approaches. Sensors.

[4] Yuan W., Lang X., Cao J., Cai Z., Zheng H. (2023). Research progress of pipeline leakage monitoring technology based on acoustic method. Oil Gas Storage Transp..

[5] Long T. (2020). Application and applicability analysis of urban drainage pipeline monitoring technology. City Town Water Supply.

[6] Sinha S.K., Knight M.A. (2004). Intelligent system for condition monitoring of underground pipelines. Comput.-Aided Civ. Infrastruct. Eng..

[7] Li T., Zheng R., Zhu J. (2006). Development status of drainage pipeline monitoring technology. China Water Wastewater.

[8] Wu T., Deng Z., Shen L., Xie Z., Chen Y., Liu C., Li Y. (2023). Research Progress on leakage monitoring technology for long distance oil pipeline. Oil Gas Storage Transp..

[9] Liu B., Liu Y., Ding K. (2022). Application of infrared imaging technology in water leakage monitoring of heating pipeline. Urban Geotech. Investig. Surv..

[10] Wu H., Zhu H., Zhu B., Qi H. (2019). Research progress and Prospect of underground pipeline monitoring based on DFOS. J. Zhejiang Univ. (Eng. Sci.).

[11] Zhang D., Huang Z., Ma Z., Yang J., Chai J. (2023). Research on Similarity Simulation Experiment of Mine Pressure Appearance in Surface Gully Working Face Based on BOTDA. Sensors.

[12] Qiu X., Zhang F., Sun Z., Jia Q., Li M. (2021). Joint monitoring method of pipeline damage based on distributed optical fiber sensing technology. Oil Gas Storage Transp..

[13] Ozevin D., Harding J. (2012). Novel leak localization in pressurized pipeline networks using acoustic emission and geometric connectivity. Int. J. Press. Vessel. Pip..

[14] Li X., Liu X., Zhang Y., Guo F., Wang X., Feng Y. (2022). Application and progress of oil and gas well engineering monitoring technology based on distributed optical fiber acoustic sensor. Oil Drill. Prod. Technol..

[15] Wang C., Liu Q., Chen D., Li H., Liang W., He Z. (2019). Pipeline leakage monitoring based on distributed optical fiber acoustic sensor. ACTA Opt. Sin..

[16] (2007). Oil and Gas Pipeline Systems.

[17] Di Y., Shuai J., Wang X., Shi L. (2013). Cause analysis and classification method of oil and gas pipeline accidents. China Saf. Sci. J..

[18] Lu H., Xu Z.D., Iseley T., Peng H., Fu L. (2023). Pipeline Inspection and Health Monitoring Technology: The Key to Integrity Management.

[19] Huang Z., He J. (2021). Research on visual monitoring system for underwater pipe network. J. Electron. Meas. Instrum..

[20] Ding C. (2022). Case study of drainage pipeline restoration project based on CCTV monitoring technology. Water Wastewater Eng..

[21] Wu W., Liu Z., He Y. (2015). Classification of defects with ensemble methods in the automated visual inspection of sewer pipes. Pattern Anal. Appl..

[22] Yuan G., Tang Y. (2017). Motion estimation of panoramic camera and 3D reconstruction of pipe network based on ASODVS. Chin. J. Sci. Instrum..

[23] Ékes C. New technologies and applications of a multi-sensor condition assessment for large-diameter underground pipe infrastructure. Proceedings of the Pipelines 2016.

[24] Falque R., Vidal-Calleja T., Valls Miro J. (2017). Defect detection and segmentation framework for remote field eddy current sensor data. Sensors.

[25] Zhou X., Liu Z. (2020). Development and teaching research of electromagnetic nondestructive monitoring technology. Electr. Drive.

[26] Sun H., Shi Y., Zhang W., Li Y. (2019). A pseudo peak removal method for far field eddy current in ferromagnetic pipes. Chin. J. Sci. Instrum..

[27] Yue Z. (2023). Research on grouting defects of bridge prestressed pipeline based on impact echo method. J. Munic. Technol..

[28] Cheng J. (2012). Acoustic Principle. J. Acoust..

[29] Okudan G., Danawe H., Zhang L., Ozevin D., Tol S. (2021). Enhancing acoustic emission characteristics in pipe-like structures with gradient-index phononic crystal lens. Materials.

[30] Juliano T.M., Meegoda J.N., Watts D.J. (2013). Acoustic emission leak detection on a metal pipeline buried in sandy soil. J. Pipeline Syst. Eng. Pract..

[31] Alobaidi W.M., Alkuam E.A., Al-Rizzo H.M., Sandgren E. (2015). Applications of ultrasonic techniques in oil and gas pipeline industries: A review. Am. J. Oper. Res..

[32] Liang J., Wu J., Liu F., Zheng M., Liu Z., Ma H. (2023). Study on defect monitoring of ultrasonic guided wave in polyurea anticorrosive pipeline. J. Mech. Strength.

[33] Yang H., Zhang H. (2023). Study on identifying defect size of duct grouting by impact echo method. Build. Struct..

[34] Iyer S., Sinha S.K. (2006). Segmentation of pipe images for crack detection in buried sewers. Computer-Aided Civil and Infrastructure Engineering.

[35] Honarvar F., Salehi F., Safavi V., Mokhtari A., Sinclair A.N. (2013). Ultrasonic monitoring of erosion/corrosion thinning rates in industrial piping systems. Ultrasonics.

[36] Lumens P.G.E. (2014). Fibre-Optic Sensing for Application in Oil and Gas Wells. Ph.D. Thesis.

[37] Tu G., Zhang X., Zhang Y., Zhu F., Xia L., Nakarmi B. (2015). The Development of an Φ−OTDR System for Quantitative Vibration Measurement. IEEE Photonics Technol. Lett..

[38] Stajanca P., Chruscicki S., Homann T., Seifert S., Schmidt D., Habib A. (2018). Detection of leak-induced pipeline vibrations using fiber—Optic distributed acoustic sensing. Sensors.

[39] Zhang Y., Yan G. (2007). Detection of gas pipe wall thickness based on electromagnetic flux leakage. Russ. J. Nondestruct. Test..

[40] Zhang J., Lian Z., Zhou Z., Song Z., Liu M., Yang K. (2022). Leakage detection in a buried gas pipeline based on distributed optical fiber time-domain acoustic wave signal. Eng. Fail. Anal..

[41] Sun Q., Li H., Fan C., He T., Yan B., Chen J., Xiao X., Yan Z. (2022). Research progress on Distributed Acoustic Wave Sensing based on Scattering-Enhanced optical fiber. Laser Optoeletronics.

[42] Barnoski M.K., Jensen S.M. (1976). Fiber waveguides: A novel technique for investigating attenuation characteristics. Appl. Opt..

[43] Healey P., Booth R.C., Daymond-John B.E., Nayar B.K. (1984). OTDR in single-mode fibre at 1.5 μm using homodyne detection. Electron. Lett..

[44] Taylor H.F., Lee C.E. (1993). Apparatus and Method for Fiber Optic Intrusion Sensing. U.S. Patent.

[45] Masoudi A., Belal M., Newson T.P. (2013). A distributed optical fibre dynamic strain sensor based on phase-OTDR. Meas. Sci. Technol..

[46] Fang G., Xu T., Feng S., Li F. (2015). Phase-sensitive optical time domain reflectometer based on phase-generated carrier algorithm. J. Light. Technol..

[47] Dong Y., Chen X., Liu E., Fu C., Zhang H., Lu Z. (2016). Quantitative measurement of dynamic nanostrain based on a phase-sensitive optical time domain reflectometer. Appl. Opt..

[48] Gorajoobi S.B., Masoudi A., Brambilla G. (2022). Polarization fading mitigation in distributed acoustic sensors based on a high-speed polarization rotator. Opt. Lett..

[49] Gabai H., Eyal A. (2016). On the sensitivity of distributed acoustic sensing. Opt. Lett..

[50] Hussels M.T., Chruscicki S., Arndt D., Scheider S., Prager J., Homann T., Habib A.K. (2019). Localization of transient events threatening pipeline integrity by fiber-optic distributed acoustic sensing. Sensors.

[51] Worsley J., Minto C., Hill D., Godfrey A., Ashdown J. Fibre optic four mode leak detection for gas, liquids and multiphase products. Proceedings of the Abu Dhabi Interinational Petroleum Exhibition and Conference.

[52] Li T., Qiao W., Li H., Sun Q., Yan Z., Liu D. (2020). Distributed acoustic sensor based sand content detection in solid-liquid two-phase flow. Proceedings of the 2020 Asia Communications and Photonics Conference (ACP) and International Conference on Information Photonics and Optical Communications (IPOC).

[53] Li T., Ai F., Hu J., He T., Li H., Sun Y., Qiao W., Yan Z., Sun Q., Liu D. (2019). Distribution Acoustic sensor based flow measurement using flow-induced vibrations. Proceedings of the 2019 18th International Conference on Optical Communications and Networks (ICOCN).

[54] Wu H., Sun Z., Qian Y., Zhang T., Rao Y. A hydrostatic leak test for water pipeline by using distributed optical fiber vibration sensing system. Proceedings of the Fifth Asia-Pacific Optical Sensors Conference.

[55] Khot S.M., Khaire P., Naik A.S. (2017). Experimental and simulation study of flow induced vibration through straight pipes. Proceedings of the 2017 International Conference on Nascent Technologies in Engineering (ICNTE).

[56] Liu Z. (2022). Research on pipeline leakage monitoring technology based on distributed optical fiber vibration sensor. Qilu Univ. Technol..

